# Cancer degronopathies and protein-fate biomarkers in drug resistance and cancer progression: signalling mechanisms, measurement and translational limits

**DOI:** 10.3389/fcell.2026.1960932

**Published:** 2026-09-25

**Authors:** Jiaxin Li, Shuhan Liu, Lu Fu, Qifu Bo, Yujie Bai, Xiangpeng Dai

**Affiliations:** 1 The First Operating Room, The First Hospital of Jilin University, Changchun, Jilin, China; 2 Clinical Medical College, Changchun University of Traditional Chinese Medicine, Changchun, Jilin, China; 3 Department of Hepatobiliary and Pancreatic Surgery, General Surgery Center, The First Hospital of Jilin University, Changchun, Jilin, China; 4 Department of Comprehensive Oncology, Affiliated Hospital of Shandong Second Medical University, Weifang, China; 5 Key Laboratory of Organ Regeneration and Transplantation of Ministry of Education, The First Hospital of Jilin University, Changchun, China; 6 National-Local Joint Engineering Laboratory of Animal Models for Human Disease, The First Hospital of Jilin University, Changchun, China

**Keywords:** biomarker, cancer progression, degron, drug resistance, protein fate, ubiquitin

## Abstract

Drug resistance remains a major barrier to durable targeted therapy, and cancer progression often reflects rewiring of signalling pathways that control protein stability. Dysregulation of the ubiquitin–proteasome system, including failure of degron-dependent substrate recognition, can stabilise oncogenic proteins, accelerate tumour-suppressor loss and generate treatment-dependent protein states. Yet clinical interpretation commonly relies on genotype or steady-state abundance, neither of which directly identifies the degradation rate or route of a specific substrate. In this critical narrative review, we use cancer degronopathy as a restricted operational term for tumour-relevant alterations in degron-dependent recognition, rather than as an established disease or diagnostic class. Through contrastive analysis of four endogenous modules (SPOP; FBXW7 versus CTNNB1; KEAP1–NFE2L2–CUL3; and VHL–HIF), together with drug-induced degradation, we examine how altered protein fate intersects with signalling pathways implicated in cancer progression, intrinsic or acquired drug resistance and therapeutic response. We trace each candidate biomarker from the initiating tumour event and substrate-specific fate change to patient-compatible measurement, analytical performance and intended clinical use. The analysis shows that SPOP effects vary with allele, substrate, lineage and assembly; FBXW7 reader defects and CTNNB1 cis-acting escape require different analytes; and pooled KEAP1-axis states and cross-isoform VHL–HIF extrapolations remain unresolved. Pharmacological degradation adds a temporal layer: vepdegestrant acts through oestrogen receptor-α degradation, whereas its FDA-authorised companion diagnostic selects patients by plasma ESR1 genotype, and serial target measurements are required to distinguish productive target loss, failed loss, recovery and downstream escape. Protein-fate measurement may be informative if it resolves a reproducible, decision-relevant state that genotype, abundance or pathway-output markers leave ambiguous. We define the experimental and clinical evidence needed to test whether such measurements can inform precision strategies to address drug resistance and cancer progression. Conclusion remain limited to the contrastive cases and United States records reviewed through 26 July 2026, with separately dated updates to selected trial records.

## 1 Introduction: clinical paradox, scope and approach

Most tumour measurements capture a molecular state at one time: sequencing identifies genomic alterations, while histopathology and proteomics record abundance and localisation. These measurements can guide clinical care without revealing how the observed state arose. Protein abundance reflects synthesis, degradation, growth dilution, trafficking and the cellular composition of the specimen. The same abundance can therefore arise from different balances of production and clearance.

Protein fate, as we use the term, is a defined kinetic or route-level property of a named protein — a rate or a path, not a level. That distinction is not merely conceptual, but it is experimentally tractable. In paired primary and metastatic organoids from a murine *Kras* ^G12D^; *Trp53* ^R172H^; *Pdx1-Cre* (KPC) pancreatic-cancer model, dynamic stable-isotope labelling of organoids (dSILO) found turnover differences that protein abundance did not consistently mirror (Ross et al., 2024). That result establishes the distinction in one model. It does not yet show that measuring turnover improves a clinical decision.

Vepdegestrant exposes the matching clinical paradox. The drug was designed to recruit oestrogen receptor-α (ERα) to cereblon (CRBN), so that ERα is degraded. Yet the pivotal VERITAC-2 trial did not select or classify patients by any measurement of ER degradation. It enrolled an all-comer population, stratified randomisation by baseline *ESR1* mutation status, and prespecified two co-primary analysis populations (Pfizer Inc. and Arvinas Estrogen Receptor and Inc, 2025; Campone et al., 2025). Its biomarker evidence concerned plasma *ESR1*. Neither ER turnover nor CRBN competence was measured. The genomic question and the protein-fate question are therefore distinct. *ESR1* status can inform a treatment-selection decision without standing in for ER degradation, while an exposure-aligned ER readout could track pharmacodynamic target loss without inheriting the selection evidence attached to *ESR1*. This separation runs through the whole review: being close to a drug’s mechanism is not, by itself, a reason to prefer a protein-state assay when a different analyte answers the clinical question at hand.

Degron biology has shown how recognition signals are created, exposed, altered or lost, and cancer studies have mapped variants that may perturb substrate stability (Mészáros et al., 2017; Tokheim et al., 2021; Zhang et al., 2025). Targeted-degrader research has, in parallel, traced molecular glues and heterobifunctional degraders from chemical mechanism towards the clinic (Békés et al., 2022; Tsai et al., 2024; Hinterndorfer et al., 2025). What remains far less settled is the step in between: how altered degradation can actually be measured in patient material and tied to a use. That step needs four things together: a named kinetic quantity, a recoverable analyte, a specified specimen and assay, and evidence matched to the decision.

We use *cancer degronopathy* as a narrow working term, not as an established disease or diagnostic class. We reserve it for a host-endogenous tumour event that alters recognition through a defined degron, or a degron-dependent state, of a named substrate, and that produces a direction-defined change in that substrate’s fate. When a reader, adaptor, E3 or deubiquitinase changes a substrate’s fate without a demonstrated dependence on a defined substrate degron, we call that degradation-circuit dysregulation instead. Drug-created recruitment is treated separately again, as pharmacological degradation. Cancer degronopathy thus denotes an evidence-defined subset of ubiquitin–proteasome system dysregulation, not a newly discovered oncogenic mechanism. An E3 alteration qualifies only when it changes degron-dependent recognition and the fate of a named substrate; pharmacological recruitment need not use that native recognition signal (Mészáros et al., 2017; Zhang et al., 2025; Békés et al., 2022; Tsai et al., 2024; Hinterndorfer et al., 2025). The contribution of this terminology is to organise the evidence required for a biomarker claim, not to replace established descriptions of E3 dysfunction or targeted protein degradation. These labels confer no diagnostic or regulatory status.

Four core modules carry the argument, each chosen to expose a different contrast: substrate and lineage dependence (SPOP); trans-acting reader loss versus cis escape (FBXW7 versus *CTNNB1*); pathway pooling (KEAP1–NFE2L2–CUL3); and isoform or specimen constraints (VHL–HIF). URI1–MDM2–p53 and USP17L2/DUB3–BRD4 extend the mechanistic range without being counted as core modules. Pharmacological degradation is kept apart, because exposure makes protein state a moving target in time.

This is a critical narrative review with a contrastive, deliberately non-exhaustive case design. We identified evidence through iterative searches of PubMed and Europe PMC, citation tracing, ClinicalTrials.gov, DailyMed and FDA records, and we checked bibliographic metadata against journal and Crossref records. A module qualified as core only when a tumour event could be linked to a named substrate by direct kinetic or route-level evidence, and when a patient-facing measurement or treatment interface exposed a distinct translational question. Prediction, binding, ubiquitylation or abundance alone did not count as causal fate evidence. We chose modules to contrast allele and lineage dependence, reader versus substrate failure, pathway pooling, isoform and specimen transfer, and treatment-time states, rather than to estimate prevalence or effect size.

We prioritised primary mechanistic and clinical reports. Protocols, registries and official regulatory records were used for study design or status, and sponsor materials only for explicitly attributed facts, never as sole evidence of efficacy, clinical validity or clinical utility. United States status is the default unless stated otherwise, and the main evidence review and regulatory-status assessment retain a cut-off of 26 July 2026. Selected trial-record updates added during revision were checked separately on 5 September 2026 and are identified below. Because selection was iterative rather than systematic, one caveat holds throughout: absence from the sources we reviewed is not evidence that an assay, use or policy does not exist. Our organising question is deliberately decision-centred. When does a substrate-specific change in degradation become a clinically interpretable measurement, and when do genotype, abundance or some other established marker already answer the question well enough? For each example we ask three things in turn: does a tumour event change the fate of a named substrate; can that change be measured in patient material; and does the assay improve a defined clinical decision? Regulatory and guideline status we treat separately, because it is specific to jurisdiction and date. A fate claim needs a causal bridge to kinetics or route. A clinically useful biomarker does not have to measure fate at all — unless fate is the very thing being asserted.

The added value of this review is to examine where established degron mechanisms do, and do not, support clinically interpretable protein-fate biomarkers. Rather than extending the mechanistic catalogue, we connect causal evidence to a feasible measurement and a defined decision. The appropriate biomarker depends on the biological question being addressed.

## 2 From degron dysregulation to a measurable protein-fate claim

A degron is the part of a substrate that the degradation machinery reads. It can be a short internal sequence, a terminal signal, a conformational surface, or a state created by modification, folding, localisation or complex assembly (Zhang et al., 2025; Wang and Zheng, 2026). BACH1 makes the point vividly: two F-box proteins recognise a quaternary-structure degron on it under oxidative stress (Cao et al., 2024). Proteome-scale screens have since named many effectors that promote degradation or stabilisation in the systems tested (Poirson et al., 2024). Discoveries like these widen the catalogue of candidate recognition mechanisms. What they do not provide is an endogenous cancer-degron map, still less a patient-level measurement of fate. A degron, its reader, and the observed abundance of its substrate must stay three separate analytical objects.

To classify a case operationally, three things must hold at once: a named substrate whose degron-dependent recognition step has changed; a direction-defined change in degradation or apparent half-life; and an explicit record of the lineage, allele, model and route actually tested. Recurrence and replication in human tissue add translational weight, but they are not part of the mechanistic definition. Large-scale mutational and reporter studies can nominate altered degrons or E3–substrate relationships, yet nomination is not causal or clinical validation (Tokheim et al., 2021; Voutsinos et al., 2026). Fusion-associated degron loss is a good illustration: it has been shown in defined constructs, in a report later corrected without changing that conclusion (Liu et al., 2021; Liu et al., 2025). Those constructs cannot, on their own, establish a lineage-specific endogenous mechanism, and the correction is part of the primary record rather than an independent replication.

The initiating event need not sit in the degron itself. A cis mutation can remove or create a recognition feature. A trans change in a reader, adaptor or E3 can shift the fate of several substrates at once. A deubiquitinase can act at the editing step, and phosphorylation, redox state, localisation or assembly can each decide whether recognition happens at all. *SPOP* shows why these distinctions matter. In defined prostate-cancer models, cancer-associated *SPOP* variants impaired degradation of full-length androgen receptor, while ubiquitylome analysis in a separate system found selective changes in substrate-site ubiquitylation and the associated substrate behaviour (An et al., 2014; Theurillat et al., 2014). That second measurement is not, in itself, a proteome-wide turnover assay. Because ubiquitin-chain architecture also controls signalling, localisation and assembly, altered ubiquitylation carries no automatic inference about degradation (Dikic and Schulman, 2023).

The evidence chain has six links: the tumour event and its context; recognition or ubiquitin editing; a protein-fate quantity; a patient readout; an analytically specified assay; and an intended use with evidence to match. The patient readout can do one of three jobs — measure fate directly, serve as a calibrated surrogate, or answer a different contextual question, as a DNA alteration may. To specify an assay you must name how it is run and on which specimen, how that specimen is handled beforehand, how the assay is calibrated and scored, where the cut-off falls, when a run counts as failed, and how well it performs.

Device authorisation, therapeutic approval and guideline status sit above all of this as separate overlays. None of them can substitute for a missing biological or measurement link.

Causal fate evidence asks for more than a predicted pairing. Deep-learning and multimodal models can prioritise degrons and E3 relationships, but their outputs stay nominations until they are tested (Hou et al., 2022; Zheng et al., 2024). A causal claim needs an interpretable change in a named substrate together with a matched perturbation that separates the proposed mechanism from its alternatives. Useful tests include repairing a cis element, comparing with matched wild type, perturbing the reader or editor, and adding catalytic or binding controls. Epistasis, route-specific interventions and an orthogonal kinetic method each test a further link. No single control supplies every inference. Independent studies that connect GSK3-dependent phosphorylation, FBXW7 recognition and altered c-MYC persistence show what combining perturbation with temporal measurement can achieve in the systems tested (Welcker et al., 2004; Yada et al., 2004). Even then, patient-assay performance and clinical use demand their own specimen and decision evidence.

Protein fate also has to be pinned to a specified quantity. Degradation rate, apparent half-life and flux through a route are distinct fate quantities. A direct fate readout estimates a specified quantity under defined conditions, accounting for synthesis, dilution and sampling time. An exposure-linked abundance trajectory instead describes a pharmacodynamic state unless kinetic calibration supports a fate interpretation. By contrast, binding, ubiquitylation, total abundance, localisation, a licensing modification and a single post-treatment time point are all non-kinetic readouts. They can be informative, but they should be read kinetically only after calibration against the quantity being claimed. We therefore reserve *protein-fate biomarker* for a direct fate measurement, or a calibrated surrogate, in a prespecified specimen, sampling context and intended use (Ross et al., 2021). The term does not stretch to cover every genomic predictor or protein assay that happens to sit near a degradation pathway.

Accelerated loss of a tumour suppressor extends the argument beyond the stabilised oncoproteins that dominate most degron examples. In *APC*-deficient, *TP53*-wild-type colorectal models, MYC-induced URI1 drove MDM2-dependent p53 loss. Manipulating URI1 changed p53 abundance, its apparent cycloheximide-chase half-life and its ubiquitylation. MG132 rescued p53, whereas blocking autophagy with chloroquine did not. Reconstitution and engineered-mouse experiments supported the same chain (Herranz-Montoya et al., 2025). Within those models the evidence ties together URI1 perturbation, MDM2 dependence, p53 ubiquitylation and apparent p53 half-life. What stays unresolved is the p53 degron itself, URI1’s enzymatic role, and p53 kinetics in patient tumours. For that reason we classify URI1–MDM2–p53 as endogenous degradation-circuit dysregulation and accelerated tumour-suppressor loss, rather than as a degronopathy or a clinical biomarker by default. At the boundary sit two neighbouring mechanisms. HPV16 E6 recruits E6AP/UBE3A to promote p53 degradation, and structural work has resolved the E6–E6AP–p53 complex (Scheffner et al., 1990; Martinez-Zapien et al., 2016). This is viral rewiring, not a host-endogenous cancer degronopathy. USP17L2, usually called DUB3, illustrates endogenous ubiquitin editing in the opposite direction. In prostate-cancer systems DUB3 bound BRD4, cut its polyubiquitylation and prolonged its persistence. Depleting DUB3 raised BRD4 ubiquitylation and shortened its apparent cycloheximide-chase half-life, and catalytic competence was required for rescue (Jin et al., 2018). These results support a causal DUB3–BRD4 relationship in the tested models. Turnover in patient tissue, specimen-assay validation and treatment selection all remain open.

Figure 1 sets out this ontology, separating the event from its recognition mechanism, the resulting fate quantity, the patient readout, the assay package and the intended use. Each transition makes a different evidential demand. A variant may flag an initiating event without naming the operative substrate. A kinetic change may be genuinely causal yet inaccessible in archived tissue. A reproducible protein measurement may still be only a static state. Even a well-performing assay may lack evidence for the decision it is meant to inform. Conversely, a genomic selector may be clinically interpretable for its own use without measuring degradation at all. The next sections apply these boundaries to four endogenous modules, before we turn to the extra, temporal constraints created by pharmacological degradation.

**FIGURE 1:**
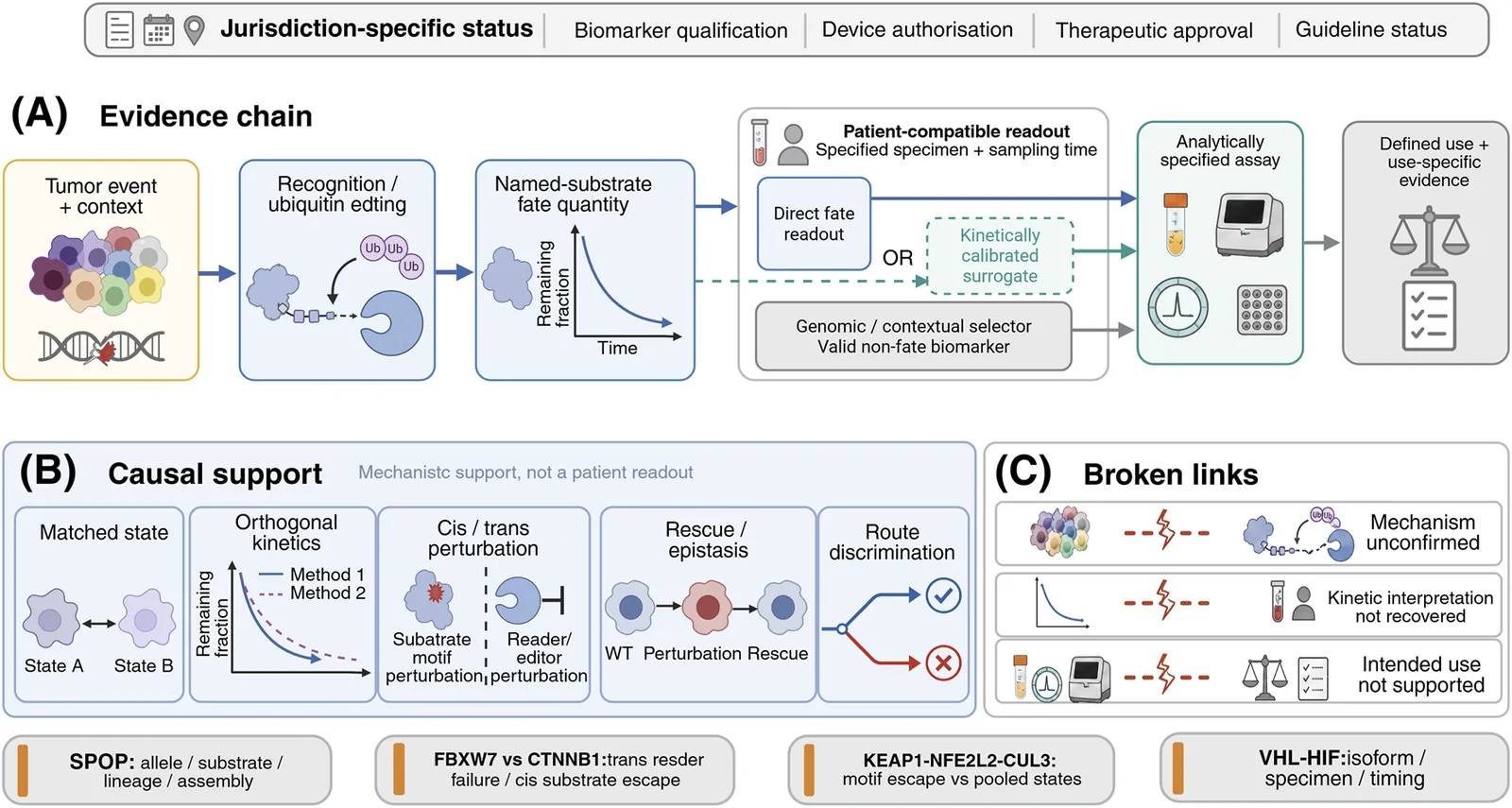
From a tumour event to a usable protein-fate biomarker. **(A)** Six distinct levels connect a tumour event and context to recognition or ubiquitin editing, a named-substrate fate quantity, a patient-compatible readout, an analytically specified assay package and a defined use with use-specific evidence. At the readout level, one branch measures fate directly or through a kinetically calibrated surrogate; the other contains valid non-fate biomarkers, such as genomic or contextual selectors, that make no fate claim. **(B)** Causal support can include a matched state, orthogonal kinetics, cis or trans perturbation, rescue or epistasis, and route discrimination. These experiments support the relevant link but are not themselves a patient readout. **(C)** Broken links mark three possible failures: the mechanism is unconfirmed; kinetic interpretation is not recovered in the intended specimen or time; or the measurement does not support the intended decision. Regulatory and guideline status is shown as a jurisdiction-specific annotation, separate from the six levels.

Terminology used throughout: protein fate denotes the degradation kinetics or clearance route of a named substrate; protein state denotes its abundance, localisation or modification at collection. A kinetic readout measures change over time under defined conditions. A calibrated surrogate is an accessible readout empirically linked to a specified kinetic quantity in matched material; calibration alone does not establish clinical utility. A pathway-state readout records downstream activity without identifying the substrate’s degradation rate.

## 3 Endogenous circuits expose different translational bottlenecks

Across the four modules a single discipline recurs: a genomic event supports a fate interpretation only after the relevant substrate, direction and context are resolved (Mészáros et al., 2017; Tokheim et al., 2021; Zhang et al., 2025; Wang and Zheng, 2026). An assay used in patient material has to preserve that lesion–substrate relationship, and genomic or pathway-state assays stay contextual comparators unless they are calibrated against the relevant kinetic quantity. The remaining questions are practical ones. Can the state be recovered in the intended specimen? Does measuring it actually improve a defined decision?

### 3.1 SPOP: allele, substrate, lineage and assembly

With *SPOP*, the genotype does not name the substrate whose fate has changed. Recurrent heterozygous missense substitutions in the *SPOP* MATH domain may define a molecular subgroup in roughly 6%–13% of primary prostate adenocarcinomas, and up to about 15% once metastatic disease is included (Barbieri et al., 2012). *SPOP* variants perturb selected interactions, so they cannot be read as a uniform drop in proteasomal activity.

The direction becomes clear only inside a specified pairing. In prostate-cancer models, MATH-domain variants impaired binding, ubiquitylation and proteasomal loss of full-length androgen receptor, and cycloheximide-chase experiments showed it persisting longer than with wild-type *SPOP* (An et al., 2014). Ubiquitin-remnant profiling in a separate system flagged a restricted set of altered sites: reduced DEK ubiquitylation went with slower inducible DEK decay, while heteromeric complexes let mutant *SPOP* suppress wild-type activity (Theurillat et al., 2014). Two further preclinical studies linked prostate-cancer *SPOP* variants to impaired BRD4 degradation, prolonged BRD4 persistence and resistance to BET inhibition (Dai et al., 2017; Zhang et al., 2017). Together these support substrate-selective loss of recognition in the models tested. The ubiquitylome, on its own, is still not a turnover measurement.

Conversely, the same gene–substrate pair can move in the opposite direction. Endometrial-cancer *SPOP* variants increased BET-protein ubiquitylation and loss, whereas the prostate-cancer variants tested stabilised BET proteins (Janouskova et al., 2017). The comparison is allele- and lineage-contingent. It is not a reciprocal experiment that isolates lineage as the cause. Assembly adds yet another source of contingency. Multiple weak *SPOP*-binding motifs increased avidity, from which the authors inferred enhanced ubiquitylation processivity, in a Gli3 model, and the avidity gain required higher-order *SPOP* oligomers rather than the dimer (Pierce et al., 2016). Higher-order *SPOP* oligomers concentrated in nuclear speckles (Marzahn et al., 2016), while multivalent *SPOP*–substrate interactions formed phase-separated compartments that promoted ubiquitylation and were disrupted by tumour mutations (Bouchard et al., 2018). Assembly therefore modifies recognition. But the finding is specific to the tested *SPOP*–substrate assembly states, and it supplies no universal quaternary-degron category.

Patient data, by contrast, remain indirect. Prostate-tumour immunohistochemistry linked *SPOP* genotype to BRD4 abundance, but measured neither BRD4 turnover nor a treatment interaction (Dai et al., 2017). A retrospective series of 72 men with *SPOP*-mutant prostate cancer described outcomes after androgen deprivation, abiraterone or enzalutamide, without a matched wild-type arm (Nakazawa et al., 2022). In a multicentre cohort of 131 *BRCA2*-altered, PARP-inhibitor-treated metastatic castration-resistant cancers, 14 tumours also carried *SPOP* alterations, but *BRCA2* allelic state and residual co-mutation could each contribute to the reported association (Orme et al., 2024). Neither cohort used a substrate-state assay. BRD4 immunoreactivity, then, is a tissue-abundance readout: not a patient half-life, and not a validated fate surrogate.

A related therapeutic programme is the randomised phase 2b study of the BET inhibitor ZEN-3694 (ZEN003694) plus enzalutamide versus enzalutamide in metastatic castration-resistant prostate cancer after abiraterone (NCT04986423; recruiting in the record checked on 5 September 2026) (ClinicalTrials.gov, 2026a). Its eligibility is not SPOP-selected, and the registered study does not validate a SPOP–BRD4 fate assay. It therefore provides a treatment-development context, not clinical confirmation of the allele-specific BET-inhibitor hypotheses.

A decisive study would first calibrate BRD4 immunoreactivity against BRD4 kinetics in allele-matched models, then test the combined variant–substrate measurement inside a single treatment comparison (Dai et al., 2017). A later clinical study would record the exact *SPOP* variant, the tumour lineage and the treatment exposure alongside the substrate measurement. A positive result would have to separate the treatment effect from allele, lineage and *BRCA2* status. A negative one would favour the simpler model — genotype plus context.

### 3.2 FBXW7 versus CTNNB1: trans-acting reader failure versus cis escape

This is a comparison of trans-acting reader failure and cis-acting substrate escape, not a shared E3–substrate pair: FBXW7 recognises substrates such as MYC and NOTCH1, whereas the CTNNB1-encoded β-catenin phosphodegron is recognised by β-TrCP/FWD1. *FBXW7* alterations and *CTNNB1* exon-3 alterations can both reduce substrate loss, yet they nominate different things to measure. An *FBXW7* lesion acts in trans, and leaves open which of several phosphorylated substrates is functionally affected. A *CTNNB1* phosphodegron lesion acts in cis, names β-catenin, and leaves open the magnitude of stabilisation and its phenotype. The assay problem therefore splits cleanly: substrate selection for *FBXW7*, allele-resolved effect size for *CTNNB1*.

For the first problem, substrate selection, MYC is the clearest case. Early perturbation and pulse-chase studies tied GSK3-dependent phosphorylation of MYC T58 to *FBXW7* binding and MYC degradation (Welcker et al., 2004; Yada et al., 2004). Later structural and cell-based work identified two cooperating diphosphorylated degrons, centred on T58/S62 and T244/T248 (Welcker et al., 2022). That same model showed that phosphorylated S62 can strengthen the higher-affinity N-terminal contact, which undercuts any simple account in which S62 must be dephosphorylated before recognition. The safe inference is cooperative, phosphorylation-dependent MYC recognition in the systems used. Neither *FBXW7* genotype nor the supporting biochemistry gives a patient-tumour estimate of MYC half-life. NOTCH1 separates cis from trans evidence inside a single study. Engineered NOTCH1 T2512A impaired *FBXW7* binding and ubiquitylation and prolonged NOTCH1 in a pulse-chase assay. Tumour-derived *FBXW7* mutants failed to recognise the NOTCH1 intracellular domain, and primary T-cell acute lymphoblastic leukaemias established that *FBXW7* mutation or homozygous deletion occurs (O'Neil et al., 2007). Each observation answers a different question: T2512A demonstrates cis escape; the *FBXW7* mutants demonstrate trans recognition failure in the tested models; the primary samples supply prevalence. Resistance of *FBXW7*-altered T-ALL lines to the γ-secretase inhibitor MRK-003 supports a preclinical treatment hypothesis, not genotype-directed resistance in patients.

The β-TrCP–β-catenin axis offers the allele-resolved cis comparison. CK1 phosphorylation at S45 primes the N-terminal sequence, and GSK3 then phosphorylates T41, S37 and S33 to complete the β-TrCP docking site (Amit et al., 2002). FWD1/β-TrCP experiments joined this region to reader binding, ubiquitylation and decay: mutation reduced binding, wild-type FWD1 promoted ubiquitylation, and dominant-negative FWD1 stabilised β-catenin in radiolabelled pulse-chase experiments (Kitagawa et al., 1999). Saturation genome editing of 342 missense substitutions across residues 31–48 returned widely varying reporter activities, and those scores told stronger from weaker β-catenin signalling in hepatocellular carcinoma (Krishna et al., 2026). The allele-level reporter is informative about functional heterogeneity. But reporter and signalling outputs are not estimates of endogenous β-catenin turnover.

Clinical evidence, for now, stays exploratory. In a prospective advanced hepatocellular carcinoma sequencing cohort of 127 patients, 31 received checkpoint-inhibitor therapy and 27 were evaluable for radiographic response; none of the 10 tumours with WNT-pathway alterations achieved clinical benefit (Harding et al., 2019). The make-up of that WNT-altered group — seven *CTNNB1*-mutant and three AXIN1-altered tumours — is recoverable from the published oncoprint and was stated explicitly in an accompanying commentary rather than in the primary report (Harding et al., 2019). The study was non-randomised, used mixed regimens, and measured neither β-catenin fate nor a treatment-by-biomarker interaction. It therefore cannot support a *CTNNB1*-specific checkpoint selector.

Clinical investigation of the WNT axis includes the phase 1/2 FOG-001 study (NCT05919264), which was enrolling at the participating centre checked on 5 September 2026. Its hepatocellular-carcinoma cohorts require a documented APC or CTNNB1 WNT-pathway-activating alteration (University of California and San Francisco, 2026). This is genomic enrichment for a pathway-directed treatment study, not validation of β-catenin turnover as a selector. The FBXW7-related γ-secretase-inhibitor resistance discussed above remains a preclinical hypothesis (O'Neil et al., 2007).

Translation should match each lesion to the substrate it nominates. For *FBXW7*, a patient-compatible package would combine the exact allele and allelic state with one lineage-relevant substrate measurement, because MYC and NOTCH1 cannot be assumed to move together (Welcker et al., 2022; O'Neil et al., 2007). For *CTNNB1*, exon-3 sequence could be paired with a calibrated β-catenin state, while reporter or transcriptional output stays a functional comparator rather than a kinetic readout (Amit et al., 2002; Kitagawa et al., 1999; Krishna et al., 2026). *APC* or AXIN alteration, lineage and regimen all offer alternative explanations for a downstream WNT signal, as the small pooled HCC composite shows (Harding et al., 2019). The intended use should be fixed before validation; for example, stratification within a specified treatment comparison. If the substrate measurement failed to track endogenous decay in allele-defined models, or to resolve outcome heterogeneity in a prospective genotype group, that would reject the added fate component.

### 3.3 KEAP1–NFE2L2–CUL3: demonstrated motif escape versus heterogeneous pooled states

Here the problem is not mechanism but pooling: one label ends up covering several different molecular states. NRF2 recognition itself is mechanistically specific. Its Neh2 domain engages a KEAP1 dimer through a high-affinity ETGE anchor and a weaker DLG latch, placing NRF2 in the CUL3–RBX1 ubiquitin-ligase complex (Zhang et al., 2004; Tong et al., 2006; Tong et al., 2007; Fukutomi et al., 2014). Cancer-associated substitutions in the two motifs weakened KEAP1 binding and polyubiquitylation in the alleles tested, and cycloheximide-chase experiments raised the apparent NRF2 half-life from about 16 min for wild type to 28–35 min for the tested variants (Shibata et al., 2008; Shibata et al., 2009). The accompanying correction changes the mutant designation to T80R and is not independent evidence. This is direct support for cis recognition escape by specified *NFE2L2* alleles, not for a shared fate consequence across KEAP1 and *CUL3* alterations.

The pooled label “KEAP1/NRF2 pathway altered” bundles together molecularly different states. An *NFE2L2* motif substitution changes the substrate’s recognition surface. A *KEAP1* variant, deletion or loss of heterozygosity acts through the reader, with consequences that depend on residual allele function and zygosity. A *CUL3* call is more remote still, until its effect on the KEAP1–NRF2 complex is actually shown. Oxidative stress, meanwhile, can activate NRF2 with no DNA event at all. NRF2 also carries two Neh6 β-TrCP recognition motifs that connect it to CUL1, and GSK3 regulation was demonstrated for one of them in the cited work (Rada et al., 2011; Chowdhry et al., 2013). In the non-small-cell lung cancer (NSCLC) samples and alleles examined, *KEAP1*-mutant cells showed greater nuclear NRF2 and target-gene expression, and ectopic wild-type KEAP1 suppressed an antioxidant response element (ARE) reporter in H838 cells more effectively than the tested tumour-derived variants (Singh et al., 2006). Nuclear abundance, transcriptional output and apparent half-life are, even so, three different analytes.

Clinical association studies have mostly pooled these distinct states together. A single-centre analysis linked pooled *KEAP1*, *NRF2* or *CUL3* alterations to shorter time to treatment failure on first-line platinum-based chemotherapy and shorter survival, with no non-platinum comparator (Jeong et al., 2020). In two lung-adenocarcinoma cohorts, the adverse PD-(L)1 outcomes tied to *KEAP1* alteration were concentrated in *KRAS*-mutant tumours, and a parallel platinum analysis showed similarly adverse outcomes, leaving prognostic and co-mutation explanations unresolved (Ricciuti et al., 2022).

An exploratory POSEIDON analysis combined *STK11* and/or *KEAP1* alterations and suggested benefit from adding tremelimumab, but it measured no protein fate and qualified no NRF2-state test (Skoulidis et al., 2024; Skoulidis et al., 2025). Its accompanying notice corrects the primary report and is not independent evidence. Retrospective analyses also found sharper outcome separation for biallelic than for monoallelic *KEAP1* inactivation in lung adenocarcinoma, including a chemoimmunotherapy subset (Zucker et al., 2025). These findings argue for resolving allele and zygosity. They do not define a regimen-independent predictor.

Therapeutically, the point sharpens further. *Keap1*-loss, *Kras*-driven lung models showed a dependence on glutaminolysis and sensitivity to glutaminase inhibition (Romero et al., 2017). The ClinicalTrials.gov record for the genotype-selected phase 2 KEAPSAKE study (NCT04265534) reports 40 enrolled participants and termination for “Lack of Clinical Benefit” (ClinicalTrials.gov, 2022). No peer-reviewed effect estimate was identified in the registry-linked and bibliographic sources we reviewed through 26 July 2026. A clinically interpretable assay would have to specify gene, allele, zygosity, *KRAS* and *STK11* context, regimen, specimen and sampling time. No routine assay in these studies resolved allelic configuration, functional pathway state and protein fate at the same time.

A separate phase 2 study is evaluating the glutamine antagonist DRP-104 after standard chemotherapy and immunotherapy in NFE2L2/KEAP1-altered NSCLC (NCT07249372; recruiting, with no results posted in the record checked on 5 September 2026) (ClinicalTrials.gov, 2026b). This study tests a genotype-associated metabolic vulnerability; it neither restores a defined NRF2 degron nor establishes a protein-fate companion diagnostic. Its activity cannot be inferred from the preclinical glutaminolysis findings or from the discontinued KEAPSAKE programme.

The appropriate measurement therefore depends on the biological question. For a question about pathway activation, a downstream NRF2 activity measurement may be enough. A stability claim must also be calibrated to turnover. Patient-facing analyses should keep gene, allele and *KEAP1* zygosity intact, and should read nuclear NRF2 or an antioxidant-response-element output as a pathway-state readout (Singh et al., 2006; Zucker et al., 2025). Redox activation, the Neh6 β-TrCP route, *KRAS* or *STK11* co-mutation and simply aggressive tumour biology all remain credible alternatives to a uniform relationship between KEAP1 loss and NRF2 half-life (Rada et al., 2011; Chowdhry et al., 2013; Jeong et al., 2020; Ricciuti et al., 2022; Skoulidis et al., 2024; Skoulidis et al., 2025). For a regimen-specific use, the decisive analysis is a treatment comparison stratified by the prespecified molecular and protein-state package. If the readout does not calibrate to direct NRF2 turnover, or the association collapses once allele, zygosity and co-mutation are resolved, it should stay a pathway-state measure, not a fate biomarker. Direct kinetic evidence for selected NFE2L2 motif variants therefore should not be assigned to pooled KEAP1, NFE2L2, CUL3 or non-genetic NRF2-activated states; their mechanisms and levels of clinical evidence remain distinct.

### 3.4 VHL–HIF: engineered mechanism, isoform and specimen boundaries

Hydroxylation-dependent HIF recognition is well established in experimental systems, but its translation to an isoform-specific kinetic assay in patient material remains unproven. In transfected cells, wild-type pVHL reduced full-length HIF-1α, and MG132 blocked that reduction (Tanimoto et al., 2000). Pro564 hydroxylation created the recognition signal, and a P564A oxygen-dependent degradation-domain fragment underwent weaker VHL-dependent ubiquitylation (Ivan et al., 2001). Full-length P564A HIF-1α was lost more slowly than wild type in an extract-based degradation time course (Ivan et al., 2001; Jaakkola et al., 2001). These results connect an oxygen-regulated cis event to HIF-1α fate. What they do not provide is a tumour-compatible kinetic assay.

Reader variants and substrate variants interrupt the chain at different points. VHL Y98N lost HIF-1α binding in the tested model, whereas C162F kept binding. Both variants nonetheless failed to reduce HIF-1α abundance (Tanimoto et al., 2000). Engineered HIF-1α P564A is a cis recognition lesion (Ivan et al., 2001). The experiments resolve causal position without supplying allele-specific HIF-1α half-lives for the *VHL* variants. Isoform transfer is limited, too, because the foundational site-specific evidence concerned HIF-1α. Experiments in 786-O cells supported oxygen- and iron-sensitive HIF-2α recognition without mapping an equivalent determinant (Ivan et al., 2001), and later work on prolyl-hydroxylase selectivity did not measure VHL binding, ubiquitylation or HIF-2α turnover (Chowdhury et al., 2016). HIF-1α kinetics, in short, cannot be assigned to HIF-2α from these data. Beyond the biochemistry lies a further layer: human genetics. A pathogenic or likely pathogenic germline *VHL* variant can establish a molecular diagnosis of VHL disease in the right clinical setting (van Leeuwaarde et al., 2025). In 53 VHL disease-associated tumours, evaluable subsets showed loss of heterozygosity, intragenic mutation or promoter methylation as routes to somatic inactivation (Prowse et al., 1997). Among the whole-exome-sequenced set of 417 sporadic clear-cell renal-cell carcinomas, 3p loss spanning VHL occurred in 91%, while promoter silencing occurred in roughly 7% and was mutually exclusive with *VHL* mutation (Cancer Genome Atlas Research Network, 2013). Population data like these do not reconstruct biallelic *VHL* configuration, or HIF fate, in any individual tumour. Static HIF abundance is also sensitive to oxygen handling and sampling time, which makes preanalytics part of the analyte definition. Belzutifan raises a separate, pharmacological question, taken up in Section 4.4.

Mechanistic evidence, isoform specificity and specimen feasibility must therefore be evaluated separately. A VHL–HIF fate claim would need isoform-specific kinetics in patient-derived material under controlled oxygen and timing, followed by calibration of whatever tissue readout is proposed (Tanimoto et al., 2000; Ivan et al., 2001; Jaakkola et al., 2001; Chowdhury et al., 2016). *VHL* allele configuration and second-allele status should stay comparators (van Leeuwaarde et al., 2025; Prowse et al., 1997; Cancer Genome Atlas Research Network, 2013). If the readout failed to agree with matched kinetics, or added nothing beyond biallelic *VHL* status and a validated pathway-state measure, it should be reported as a static HIF-state measurement rather than a validated fate surrogate.

## 4 Treatment-time protein states: induced degradation and an adjacent inhibitor boundary

Drug-induced recruitment depends on the administered compound and exposure, unlike host-endogenous recognition defects. Productive target loss, failed loss, recovery after an initial fall and downstream escape despite maintained loss cannot be told apart from baseline genotype or abundance. Telling them apart needs exposure-aligned target or neosubstrate measurements and, for recovery, serial sampling. These states describe observed trajectories. They should not be assigned when the time points that would establish them are missing. Figure 2 gathers the exposure-aligned states and the measurements each one calls for.

**FIGURE 2:**
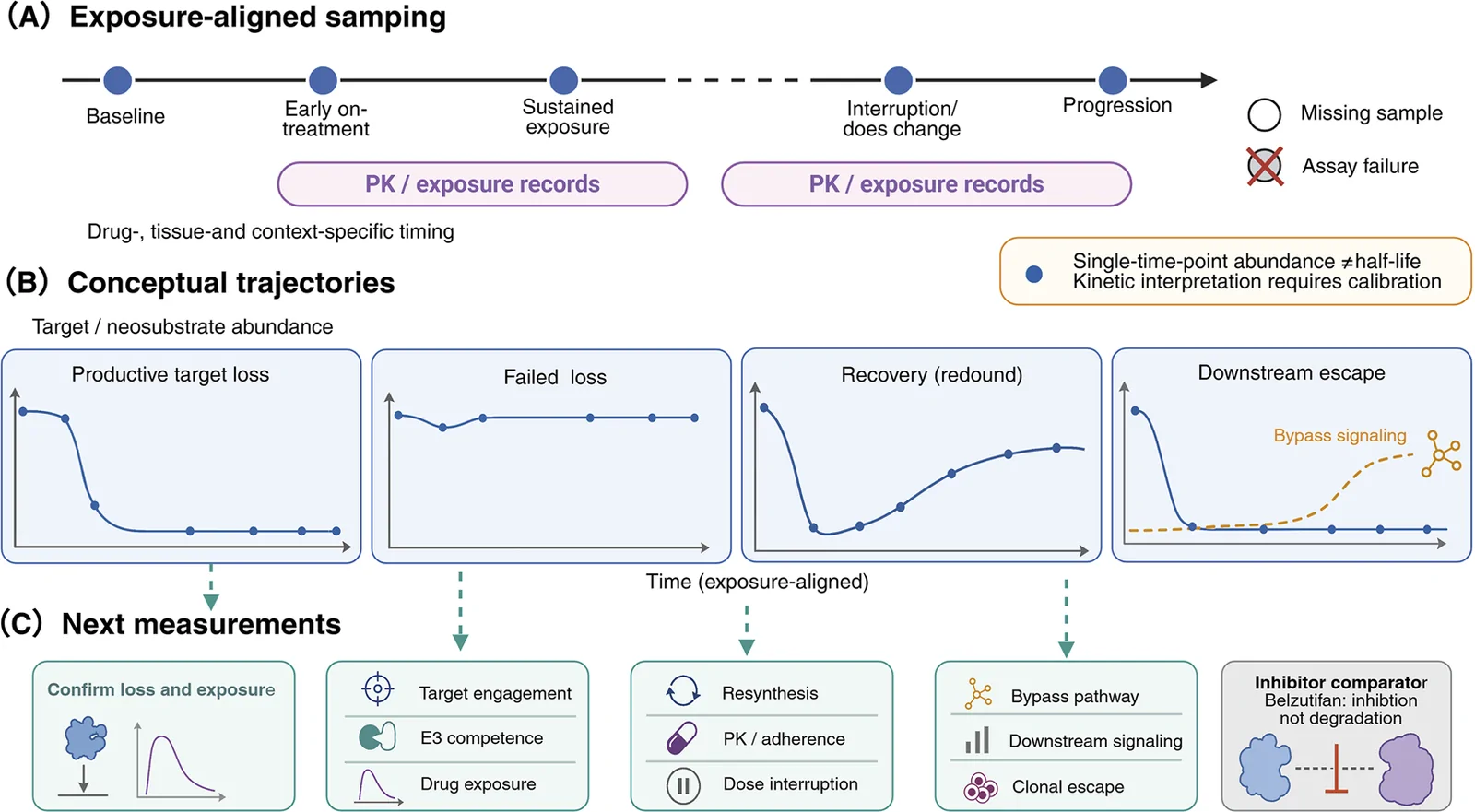
Protein fate under treatment is an exposure-aligned trajectory. **(A)** The conceptual sampling axis aligns baseline, an early on-treatment point, sustained exposure, interruption or dose change, and progression with pharmacokinetic information. The schedule is adapted to the drug, tissue and clinical setting; it is not a universal biopsy prescription. **(B)** Serial measurements distinguish four candidate states: productive target loss, failed recruitment or failed loss, recovery after an initial fall (labelled “Recovery/resynthesis” in the figure), and downstream escape despite maintained loss. Here, rebound is synonymous with recovery, whereas resynthesis is a possible mechanism requiring separate evidence; failed recruitment is likewise a candidate explanation for failed loss, not a conclusion from abundance alone. **(C)** Each pattern directs a different next measurement, including E3 competence, target engagement, synthesis, exposure and pathway escape. Target abundance at one time point is interpreted as pharmacodynamic evidence unless a calibrated temporal design supports a stronger kinetic claim. Assay failures and missing sampling points remain visible. The trajectories do not establish prediction, treatment benefit or clinical utility, and belzutifan is excluded from the degradation trajectories and shown separately in panel C as an inhibitor comparator because it blocks HIF-2α dimerisation without inducing degradation (Dail yMed, 2026).

### 4.1 Drug-created recognition

The cleanest examples are drugs that build a recognition surface where none existed. Lenalidomide is the classic case. Structural work defined the DDB1–CRBN–thalidomide complex (Fischer et al., 2014), and two independent studies showed that drug-bound CRBN recruits IKZF1 and IKZF3 to CRL4–CRBN for ubiquitylation and degradation in myeloma models (Krönke et al., 2014; Lu et al., 2014). Cereblon E3 ligase modulators (CELMoDs) favour a closed CRBN conformation that governs neosubstrate recruitment and efficacy in the systems tested (Watson et al., 2022). The causal unit, then, is the specified drug, ligase, neosubstrate and exposure, taken together. Baseline CRBN expression cannot stand in for an exposure-timed measurement of IKZF1 or IKZF3 loss.

Indisulam and E7820 build recognition through a different adaptor. In cell-line models, DCAF15 recruited RBM39 to CRL4–DCAF15, causing RBM39 degradation and mis-splicing, and DCAF15 expression tracked with sensitivity in the reported analyses (Han et al., 2017). Independent work found degradation of CAPERα, now called RBM39, after E7820 exposure (Uehara et al., 2017). These data support the DCAF15–RBM39 mechanism in the tested systems. What they do not support is transferring IKZF-based assays or sensitivity thresholds across from CRBN-directed therapy.

Mezigdomide provides an exploratory patient pharmacodynamic observation. In the phase 1/2 study, maximum Aiolos degradation in peripheral blood appeared within 3 h at daily doses above 0.3 mg, with the signal reported through 24 h (Richardson et al., 2023). This is an exploratory, exposure-linked blood readout, not a tumour or marrow selector, and not evidence of transfer across CELMoDs or immunomodulatory drugs. Pretreatment formalin-fixed, paraffin-embedded (FFPE) marrow immunohistochemistry for CRBN, IKZF1 and IKZF3 did not separate lenalidomide-sensitive from resistant cases among 23 evaluable samples (Dimopoulos et al., 2019). In a separate 44-patient pomalidomide–dexamethasone series, a data-derived lowest quartile of IKZF1 RNA in CD138-selected marrow was associated with no responses, but the array-based threshold lacked external validation (Zhu et al., 2014). Static expression and *post hoc* transcript associations do not, between them, demonstrate neosubstrate loss.

An exposure-timed fate measurement could support an adaptive pharmacodynamic or treatment-continuation use only if a prespecified result improved a defined on-treatment decision beyond baseline expression and clinical covariates. A pretreatment selection claim would need a separate baseline assay and an appropriate treatment-by-biomarker analysis. Exposure, not baseline expression, makes these readouts interpretable.

### 4.2 Vepdegestrant: mechanism, target loss and genomic selection

Vepdegestrant is where mechanism and selector visibly come apart. As of 26 July 2026 it was an oral heterobifunctional degrader approved in the United States for its labelled *ESR1*-mutated population (DailyMed, 2026). In ER-positive breast-cancer models, inactive-ligase controls, proteasome blockade and ligand competition together supported CRBN-dependent ERα loss (Gough et al., 2024). These experiments establish drug-created recognition and target loss in the models tested. Model concentration–response data, however, provide no clinical threshold.

Biomarkers of endogenous dysregulation cannot therefore be assumed to transfer directly to proteolysis-targeting chimaeras (PROTACs) or molecular glues. A native degron defect need not prevent drug-created recruitment, and preserved native turnover does not establish competence of the recruited E3. PROTAC activity requires productive drug–target–E3 assembly, whereas molecular glues create or stabilise a drug-dependent ligase–neosubstrate interface (Fischer et al., 2014; Krönke et al., 2014; Lu et al., 2014; Watson et al., 2022; Han et al., 2017; Uehara et al., 2017; Gough et al., 2024). Endogenous genotype and baseline abundance may remain contextual covariates, but the assay and threshold require validation for the specific compound, recruited ligase, target, tissue and exposure. Pretreatment selection and on-treatment pharmacodynamic monitoring are separate intended uses.

Clinical evidence for ER target modulation is limited to an exploratory paired-biopsy analysis, reported secondarily (Gough et al., 2024). Because that paper is not the primary clinical report for the analysis, we do not use its numerical estimate here. The observation is provisional evidence that exposure-timed ER abundance can be collected, not an estimate of ER degradation rate or half-life, and not evidence of prediction or utility. Presurgical pharmacodynamic measurement does have precedent: a window-of-opportunity comparison of the oral SERD AZD9496 with fulvestrant in newly diagnosed breast cancer (Robert et al., 2020). That comparator supports the feasibility of timed ER assessment. It does not supply a kinetic bridge for vepdegestrant.

VERITAC-2 randomised an all-comer population irrespective of *ESR1* status and tested progression-free survival in two prespecified co-primary populations: participants with *ESR1*-mutated disease and all randomised participants. A graphical multiple-testing strategy controlled multiplicity rather than imposing a fixed-sequence hierarchy (Pfizer Inc. and Arvinas Estrogen Receptor and Inc, 2025; Campone et al., 2025). Section 6 details this design and its implications for biomarker interpretation.

The prespecified *ESR1*-mutant analysis and the regulatory selection strategy used baseline plasma *ESR1* status, not an assay of ER loss. Plasma *ESR1* is therefore a genomic selector, not a protein-fate readout. The secondary paired-biopsy abundance observation and the plasma selector answer different questions and cannot validate one another. Section 6 examines the companion-diagnostic bridge and the authorisation record. An ER fate assay would need to improve response or resistance interpretation beyond *ESR1* status and standard covariates.

### 4.3 Failed loss, recovery and downstream escape

Resistance phenotypes become interpretable only once the target state is actually measured. Three lenalidomide-selected myeloma models with CRBN deletion, mutation or reduced expression showed abrogated or substantially reduced endpoint IKZF1/3 loss, and CRBN restoration recovered lenalidomide sensitivity (Zhu et al., 2019). These support failed loss. XG1LenRes told a different story: it kept CRBN and endpoint IKZF1/3 loss despite resistance, alongside IL-6-STAT3 signalling and a truncated IRF4 programme (Zhu et al., 2019). That pattern supports probable downstream escape in that model, while resistant-state kinetics, patient frequency and a clinical classifier all remain untested.

Recovery, also termed rebound, denotes an observed increase after initial target loss. Resynthesis is one possible cause, not a synonym. The Figure 2 label “Recovery/resynthesis” therefore names the trajectory and a candidate mechanism, respectively. Exposure-aligned serial samples establish recovery; a resynthesis claim additionally requires evidence of new protein production. A resistant endpoint alone cannot establish recovery. An alternative-E3 experiment tests something else again. A DCAF1-recruiting degrader produced BRD9 loss in VHL-null 786-O cells under conditions where a VHL-recruiting comparator was impaired (Schröder et al., 2024a; Schröder et al., 2024b). The associated author correction changes a methods interval from 4 days to 4 hours and repairs chemical structures. It is not independent evidence. The experiment shows that E3 availability matters in that system, not acquired resistance in patients, and not a general rescue strategy for CRBN-resistant disease.

A clinical trajectory classifier would require prespecified sampling windows, exposure records, a validated target assay and an outcome model that actually separates the three states. Early-loss, interruption and progression samples would have to be read as one linked series, since an isolated endpoint cannot establish recovery. Its value would be falsified if serial target measurements neither classified resistance reproducibly nor improved treatment decisions over baseline genotype and routine response assessment.

### 4.4 Belzutifan: an adjacent HIF-2α inhibitor boundary

Belzutifan provides a clinically established HIF-2α-inhibitor comparator, not an induced degrader. The June 2026 United States label states that it binds HIF-2α and blocks its interaction with HIF-1β (also known as ARNT), reducing HIF-2α target-gene transcription (Dail yMed, 2026). Its mechanism recruits no E3 ligase and induces no HIF-2α destruction. Belzutifan therefore serves as a comparator for pathway inhibition without induced degradation.

Clinical activity and regulatory approval answer treatment questions without validating any HIF fate assay. A single-group study reported activity in VHL disease-associated renal-cell carcinoma (Jonasch et al., 2021). A randomised trial compared belzutifan with everolimus in advanced clear-cell renal-cell carcinoma after prior immune-checkpoint and antiangiogenic therapy (Choueiri et al., 2024). A separate single-group phase 2 study preceded the 2025 United States approval in advanced pheochromocytoma or paraganglioma (Jime et al., 2025; U.S. Food and Drug Administration, 2025a). In 2026, the United States approved adjuvant belzutifan plus pembrolizumab for renal cell carcinoma with a clear cell component at intermediate-high or high risk of recurrence following nephrectomy (Choueiri et al., 2026; U.S. Food and Drug Administration, 2025b). Across all of this, the June 2026 United States label and the FDA companion-diagnostic list (last updated 5 February 2026; accessed 26 July 2026) identified no VHL/HIF protein-fate companion diagnostic for these indications (Dail yMed, 2026; U.S. Food and Drug Administration, 2025c).

A VHL/HIF measurement should be proposed only for a defined decision that current clinical and pathway-state variables leave unresolved. It would need an isoform-specific analyte, controlled specimen handling and a bridge to turnover. If those data failed to improve patient selection or pharmacodynamic interpretation, the proposed assay would have no demonstrated added value for that use, irrespective of belzutifan’s established therapeutic activity.

## 5 Recovering fate from viable models and clinical specimens

A protein-fate claim changes meaning the moment the measurement moves from a viable model to a clinical specimen. In living material, a time-resolved experiment can estimate disappearance, replacement or flux. A fixed section, a frozen aliquot or a plasma sample records only a state at collection. That state can still be useful, but it may be read as fate only after it has been calibrated against the matching kinetics for the named substrate, the biological context and the sampling window. The bridge also has to separate degradation from synthesis, growth dilution, trafficking, exposure and shifts in cell composition. Table 1 therefore states the strongest inference each measurement class permits, rather than ranking technologies (Ross et al., 2024; Ross et al., 2021). Translation-arrest chases estimate how a pre-existing protein pool declines once synthesis is blocked. Their slope is an apparent decay under those conditions, not an unperturbed degradation constant. Radiolabelled pulse-chase experiments instead follow a labelled cohort, and can support a substrate-specific decay estimate when labelling, chase efficiency and compartmental exchange are controlled (Ross et al., 2021; Kitagawa et al., 1999). HaloTag pulse-chase adds an orthogonal cohort-labelling comparator, though that study does not validate cycloheximide itself (Merrill et al., 2019). Whatever the design, the quantity to be estimated should be named before any curve is fitted — apparent half-life, fractional replacement, degradation rate, or another explicitly modelled quantity.

**TABLE 1:** From kinetic reference to patient-compatible readout: what each measurement can and cannot establish.

Measurement class	Primary measurand	Maximum supported inference	What it does not establish or key confounders	Bridge and controls required for the stated claim	Specimen and timing boundary
Translation-arrest or radiolabelled pulse-chase kinetics	Decline of a pre-existing or labelled substrate pool over time	Apparent decay under arrest, or cohort-specific decay with an interpretable chase	Unperturbed degradation if synthesis is arrested; synthesis, dilution, toxicity and compartment exchange	Matched state; viability and perturbation controls; orthogonal cohort method; route perturbation	Viable cells or tissue; dense, prespecified time course
Dynamic isotope labelling and model-based turnover	Label incorporation or replacement with precursor and abundance data	Model-based turnover, synthesis or degradation quantities supported by the fitted design	Identifiability outside the model; transfer from murine organoid, cell or animal systems to routine human specimens	Precursor enrichment; total abundance; growth or dilution; raw trajectories; uncertainty	Viable models or *in vivo* sampling compatible with serial labelling
Fluorescent timer, pulse tag or degron-transfer reporter	Relative protein age, tagged-cohort loss or motif effect in a construct	Relative dynamics or motif sufficiency in the tested construct	Endogenous full-length fate; effects of maturation, tag position, expression and incomplete processing	Full-length and endogenous confirmation; matched construct; orthogonal kinetics	Viable engineered systems; timing constrained by tag and reporter kinetics
Protein abundance and localisation in clinical specimens	Total or compartment-specific protein signal at collection	Patient-compatible protein state and spatial distribution	Degradation rate or route; separation from synthesis, dilution, trafficking and composition	Analyte-specific validation; tumour-cell scoring; kinetic calibration if a fate claim is made	Frozen or FFPE tissue at a recorded collection time and treatment state
Licensing post-translational modification (PTM) or K-ε-GG peptide	Modification or diglycine remnant at a defined site	Presence or relative change of a defined modification or ubiquitylated site at collection	Cognate E3, modifier, chain topology, route, occupancy without both pools, or turnover	Matching total peptide; recovery and interference studies; perturbation and kinetic calibration	Matrix- and processing-specific; non-detection and collection time retained
Exposure-timed serial target abundance with pharmacokinetics	Target or neosubstrate abundance across treatment times and exposure	Pharmacodynamic loss, failed loss, recovery or maintained loss pattern	Half-life without calibration; prediction, benefit or utility from target loss alone	Baseline and serial measurements; pharmacokinetics (PK); assay quality control (QC); synthesis, E3 and downstream measures chosen by pattern	Drug-, tissue- and context-specific sampling; no universal schedule
Genomic, transcriptomic, spatial or single-cell contextual adjuncts	Alteration, RNA, epitope abundance, composition or localisation	Initiating lesion, clonal context, cellular composition and spatial localisation	Intracellular substrate degradation, half-life or flux	Variant- and matrix-specific validation; tumour fraction; integration with a separately calibrated fate readout when claimed	Tissue, plasma or fixed spatial material; compartment and collection time explicit

Representative sources by row: translation arrest or pulse-chase (Ross et al., 2021; Kitagawa et al., 1999); dynamic isotope labelling (Ross et al., 2024; Mathieson et al., 2018; Savitski et al., 2018; Alamillo et al., 2025; Li et al., 2025); reporter systems (Tokheim et al., 2021; Merrill et al., 2019; Khmelinskii et al., 2012; Khmelinskii et al., 2016); clinical-specimen abundance (Haines et al., 2025; Tüshaus et al., 2025; Berkovska et al., 2026; National Cancer Institute, 2026; Carr et al., 2014; Goldsmith et al., 2024; Addona et al., 2009a; Addona et al., 2009b; Gandhi et al., 2014); PTM, or K-ε-GG (Dikic and Schulman, 2023; Udeshi et al., 2012; Mertins et al., 2013; Udeshi et al., 2013); exposure-timed target abundance (Richardson et al., 2023; DailyMed, 2026; Gough et al., 2024; Robert et al., 2020; Zhu et al., 2019); contextual adjuncts (Deveson et al., 2021; Silvestro et al., 2026; Stoeckius et al., 2017; Rodriques et al., 2019; Merritt et al., 2020).

Cis repair, matched wild type and variant, reader, E3 or DUB, perturbation, rescue, epistasis and orthogonal kinetics can support causality when appropriate. They are supporting experiments, not a standalone patient readout.

Dynamic isotope labelling pulls apart the layers that static abundance runs together. Label incorporation, precursor enrichment, total protein abundance and cellular growth or dilution each enter the kinetic model separately, and the fitted uncertainty should stay visible beside the raw trajectory (Mathieson et al., 2018; Savitski et al., 2018). Heavy-water and related *in vivo* approaches carry this logic across tissues, and dSILO has shown turnover changes in paired primary and metastatic organoids from a murine KPC pancreatic-cancer model (Ross et al., 2024; Alamillo et al., 2025; Li et al., 2025). These studies establish that model-based turnover measurements are feasible in their stated systems. Turning that into a routine human-tumour assay is a separate step.

What can a reporter actually establish? Something more local. A transferred degron can test whether a sequence is enough to alter a construct, and a fluorescent timer can estimate relative protein age (Tokheim et al., 2021; Khmelinskii et al., 2012). Maturation kinetics, tag orientation, expression level, incomplete proteasomal processing and loss of the native context can each move the signal (Khmelinskii et al., 2012; Khmelinskii et al., 2016). A reporter result therefore needs confirmation in the full-length protein and, where feasible, at the endogenous locus with an orthogonal kinetic method. Agreement between variants of the same construct is technical replication. It is not evidence that the endogenous substrate shares the same fate.

Once the specimen is processed, a further boundary appears. Viable slices or organoids can support short perturbations or labelling, but excision and culture establish a new physiological state. Rapid freezing preserves a molecular snapshot; FFPE preserves morphology and assay-dependent epitopes; plasma samples a different compartment altogether. Recent FFPE proteomic and phosphoproteomic studies show real analytical depth in fixed tissue (Haines et al., 2025; Tüshaus et al., 2025; Berkovska et al., 2026). By design, though, a cross-sectional profile cannot reconstruct the intracellular rate that preceded it without a kinetic bridge. Collection time, cold ischaemia, fixation, oxygenation, treatment carry-over and tumour content must therefore travel with the result, as variables that can change its interpretation (National Cancer Institute, 2026).

If calibration is to work, it has a natural order. First establish the kinetic quantity and the causal perturbation in lineage-matched viable material. Then test a prespecified proximal readout in paired viable, rapid-frozen and FFPE preparations, adding plasma where it is relevant. Lesion, sampling time, cold ischaemia, fixation, tumour fraction, exposure and assay outcome stay attached to every observation (National Cancer Institute, 2026). Targeted peptide assays need fit-for-purpose development, and an immunohistochemical result is valid only for its defined antibody, protocol, scoring method and specimen context (Carr et al., 2014; Goldsmith et al., 2024). This design tests whether interpretation survives both the viable-to-fixed transition and the move between specimen types.

Targeted proteomics and immunohistochemistry answer complementary questions. Internal-standard peptide measurements can quantify a prespecified analyte, while immunohistochemistry preserves cell identity and localisation. Neither platform inherits cross-analyte validity from a successful precedent. A multi-site plasma multiple-reaction monitoring (MRM) study supports the feasibility of reproducible targeted measurements, not a tissue assay for a degron circuit (Addona et al., 2009a; Addona et al., 2009b). By the same token, standardisation work on cereblon reagents cannot validate NRF2, β-catenin, HIF or ER staining (Gandhi et al., 2014). Each proposed surrogate needs its own matrix-specific recovery, interference, precision, reportable range, scoring and failure criteria, and then calibration against matched kinetics for the exact substrate (Carr et al., 2014; Goldsmith et al., 2024).

Recognition-proximal measurements shorten the mechanistic distance without closing the chain. A licensing phosphorylation or hydroxylation can record a state that recognition requires. A lysine ε-glycylglycine (K-ε-GG) remnant peptide records that modification at collection (Udeshi et al., 2012; Mertins et al., 2013; Udeshi et al., 2013). Quantifying it relative to the matching total peptide helps control for total-protein abundance, but site occupancy needs quantitative resolution of the modified and unmodified pools. The remnant on its own leaves the modifier, the cognate E3, chain architecture, route and turnover rate all unidentified (Dikic and Schulman, 2023; Udeshi et al., 2012; Mertins et al., 2013; Udeshi et al., 2013). Non-detection, moreover, may reflect biology, peptide recovery or analytical sensitivity. Fresh-frozen performance cannot be assumed to hold in FFPE, and a change between baseline and treatment samples stays conditional on specimen handling, tumour content and exposure.

Genomic and contextual assays cover neighbouring uncertainties. Tissue next-generation sequencing (NGS) can define an initiating allele and, subject to coverage and tumour purity, its allelic context. Plasma circulating tumour DNA (ctDNA) can detect or follow a lesion, but a negative result has to be read against input, variant-specific performance and evidence that tumour-derived DNA was present at all (Deveson et al., 2021; Silvestro et al., 2026). Spatial transcriptomics maps RNA; cellular indexing of transcriptomes and epitopes by sequencing (CITE-seq) pairs transcripts with affinity-defined epitopes in dissociated cells; fixed-tissue spatial assays localise selected proteins and RNA (Stoeckius et al., 2017; Rodriques et al., 2019; Merritt et al., 2020). These measurements can expose composition, localisation and clonal context. Even so, DNA and RNA do not measure intracellular substrate fate, and epitope abundance stays a state until it has been kinetically calibrated.

What can actually be recovered is itself part of the claim. Access, consent, lesion choice, tissue adequacy, tumour fraction and assay success all decide who ends up in the evaluable set. Reporting only the successful pairs can hide the selection created by failed or missing specimens. Preanalytical excursions can move a signal, and progression samples add anatomical and clonal differences (National Cancer Institute, 2026; Goldsmith et al., 2024). A defensible study therefore reports every attempted assay, and assigns each non-result a specific label: missing specimen, analytical failure, indeterminate result, or prespecified biological non-detection. Table 1 places these constraints beside the maximum inference for each readout. A patient-compatible assay is judged by what it measures in its intended specimen and time, not by how close it sits to an appealing mechanism.

Clinical feasibility remains a major limitation. Translation-arrest and reporter assays require viable or engineered material; isotope-based methods require controlled labelling, precursor-enrichment measurements, serial sampling and specialised mass spectrometry (Ross et al., 2024; Ross et al., 2021; Merrill et al., 2019; Mathieson et al., 2018; Savitski et al., 2018; Alamillo et al., 2025; Li et al., 2025). These are primarily kinetic reference methods, not established routine oncology tests. Archived FFPE tissue cannot be labelled retrospectively, and *ex vivo* culture may alter the state being estimated. Serial biopsies add procedural and tissue-access constraints, while blood cannot be assumed to represent tumour target turnover. A practical development strategy is therefore to calibrate a tissue readout in matched patient-derived material and then test its reproducibility across laboratories, including handling tolerances, scoring, failure rates and predefined thresholds (National Cancer Institute, 2026; Carr et al., 2014; Goldsmith et al., 2024; Addona et al., 2009a; Addona et al., 2009b; Gandhi et al., 2014). Exposure-timed target abundance may support pharmacodynamic monitoring, but not a degradation-rate claim without calibration.

Before any external validation, the expected relation between the specimen readout and the kinetic reference should be written down in advance: its functional form, its uncertainty, and its acceptable preanalytical range. A pooled correlation may be driven by tumour composition or substrate abundance, so it should be tested inside the relevant allele and lineage. Replicate sections gauge local precision. Material from different lesions tests biological transportability. Development and validation cohorts must be separated by patient, and clustering kept whenever one patient contributes several samples. If adjusting for tumour content, synthesis or exposure erodes the association, the assay should be reported as a state measurement rather than a fate surrogate (Ross et al., 2021; National Cancer Institute, 2026; Carr et al., 2014; Goldsmith et al., 2024).

## 6 From an analytically specified assay to clinical use

Clinical interpretation turns on the exact population, analyte, specimen, sampling time, assay, treatment comparison and endpoint; changing any one changes the evidentiary question. Analytical performance concerns how the measurement behaves. Clinical validity links the result to the specified biological or clinical state or outcome, in the intended population. Clinical utility asks a harder question again: does deploying the testing strategy change management, or net benefit? Biomarker qualification, device authorisation and therapeutic-product approval each follow a different regulatory record. The Biomarkers, EndpointS, and other Tools (BEST) Resource supplies useful terminology, but it confers no achieved status on a candidate (FDA–NIH Biomarker Working Group, 2016). The vepdegestrant record is specific to one assay and one use. P200010/S010 documents an earlier plasma *ESR1* application for the Guardant360 companion diagnostic (CDx), and provides platform precedent, not exact-use evidence for vepdegestrant (U.S. Food and Drug Administration, 2023a). The final device for vepdegestrant is Guardant360 CDx under premarket approval (PMA) supplement P200010/S025, with the new drug application review supplying the associated bridge (U.S. Food and Drug Administration, 2026a; U.S. Food and Drug Administration, 2026b). As of 26 July 2026 the authorised package was a specified plasma NGS test for the labelled *ESR1* mutation scope in the United States (U.S. Food and Drug Administration, 2025c). Assay name, plasma specimen, variant scope and treatment use belong together, and none of them can be widened by analogy.

Vepdegestrant’s mechanism and its clinical selector involve different analytes. The drug binds ER and CRBN and induces CRBN-mediated polyubiquitination and proteasomal loss of ER. The authorised clinical selector measures plasma *ESR1* genotype (DailyMed, 2026; U.S. Food and Drug Administration, 2026a; U.S. Food and Drug Administration, 2026b; U.S. F ood and Drug Administration, 2026). It does not measure ER abundance, ER degradation, CRBN competence or ternary-complex formation. Keeping the two apart avoids two opposite errors: recasting the pharmacology as a genomic measurement, or demanding that a genomic selector somehow measure protein fate. A separate ER-fate assay would need its own analyte, specimen, time, calibration and intended use. VERITAC-2 enrolled an all-comer population stratified by *ESR1* status and assessed progression-free survival in two co-primary populations: participants with *ESR1*-mutated disease, and all randomised participants (Campone et al., 2025). Its amended testing procedure allocated alpha across the two co-primary populations using a graphical multiple testing strategy, controlling the one-sided family-wise error rate at 2.5%, testing each at a one-sided level of 0.01875 and recycling alpha between them, rather than placing the *ESR1*-mutated population in a fixed sequence. This design is not biomarker-positive-only enrichment, and the prespecified subgroup should not be described as *post hoc*. Nor can it be converted into an interaction study after the fact. No formal treatment-by-*ESR1* interaction test was reported in the primary trial publication, so separate significance results cannot establish that interaction.

The numbers themselves are worth stating precisely. Among 270 participants with *ESR1*-mutated disease, median progression-free survival was 5.0 months with vepdegestrant and 2.1 months with fulvestrant. The primary publication reported a hazard ratio of 0.58. The FDA analysis reported 0.57 (Campone et al., 2025; U.S. Food and Drug Administration, 2026b; U.S. F ood and Drug Administration, 2026). In all randomised participants, the hazard ratio was 0.83, with P = 0.07 (Campone et al., 2025). Each value is kept with its source. These findings establish efficacy in the stated populations. They do not assess ER degradation, and they do not test prospective use of the final device across all participants.

FDA-supported clinical validity for the labelled companion-diagnostic use rested partly on a retrospective bridge between the final device and the clinical-trial testing route, rather than on prospective final-device testing of the full randomised cohort (U.S. Food and Drug Administration, 2026b). As of 26 July 2026, the summary of safety and effectiveness data for premarket approval supplement P200010/S025 had not been published, so the exact bridging cohort size and positive and negative percent agreement could not be verified from public records. The bridge links the final Guardant360 CDx result to the trial testing route for that use. Because testing routes were mixed, it neither represents prospective final-device testing of the full cohort nor constitutes an independent validation cohort. This is a gap in the public documentation reviewed here, not evidence that the bridge was inadequate.

On 1 May 2026, FDA approved vepdegestrant for adults with ER-positive, HER2-negative, *ESR1*-mutated advanced or metastatic breast cancer, detected by an FDA-authorised test, after progression on at least one line of endocrine therapy (U.S. F ood and Drug Administration, 2026). On the same date, Guardant360 CDx received authorisation for the specified plasma *ESR1* selection use under P200010/S025 (U.S. Food and Drug Administration, 2026a). FDA characterised vepdegestrant as a heterobifunctional protein degrader (U.S. F ood and Drug Administration, 2026). On the FDA-authorised record reviewed here it is the first drug of this class to receive United States approval. The “first-in-class” and “first PROTAC” framings come from sponsor and secondary sources rather than from the Agency’s own documents (U.S. Food and Drug Administration, 2026b; U.S. F ood and Drug Administration, 2026). The statement is specific to FDA approval and classification, and makes no claim to global priority among targeted protein degraders.

Clinical validity and device authorisation, however, do not quantify the incremental net benefit of a testing strategy. VERITAC-2 did not directly compare use of the final device with no testing, or with an alternative testing strategy (Campone et al., 2025; U.S. Food and Drug Administration, 2026b). The trial therefore cannot estimate the benefit, harm or resource consequences of deploying that test strategy. This is a design-specific evidence gap, not a negative utility result. The same discipline applies to the exploratory paired-biopsy ER measurements: a pharmacodynamic signal can be informative even though it was never used to assign treatment.

The intended decision dictates the trial design. A treatment-effect-modification claim needs prespecified comparison across biomarker strata and an appropriate interaction analysis. A biomarker-positive-only trial estimates the treatment effect in the enrolled positive population, but cannot estimate the corresponding negative-population effect (U.S. Food and Drug Administration, 2019). A prospective–retrospective bridge can support a defined use when the assay, eligible variants, population, comparator, endpoints, missing-data rules and analysis are all prespecified. For a pharmacodynamic use, exposure-aligned serial target measurements may establish a treatment-linked state. Predictive, kinetic and test-guided claims each require their own separate evidence.

The three regulatory tracks stay distinct. United States biomarker qualification concerns a stated drug-development context of use. Device authorisation concerns a specified assay and intended use, and companion-diagnostic authorisation ties the device to a defined therapeutic decision (U.S. Food and Drug Administration, 2014; U.S. Food and Drug Administration, 2018). Therapeutic-product approval follows a different record again. Belzutifan illustrates the split: its approvals establish therapeutic-product status, yet the June 2026 United States label and the FDA companion-diagnostic list (last updated 5 February 2026; accessed 26 July 2026) identified no VHL/HIF protein-fate companion diagnostic for those indications (Dail yMed, 2026; U.S. Food and Drug Administration, 2025c).

Mechanistic proximity, finally, is no substitute for comparative performance. A protein-state component can add information when turnover and abundance diverge, but it can just as easily add missing values, unstable calibration, or no decision-relevant separation at all (Ross et al., 2024). A candidate fate measurement should therefore be compared externally with genotype, pathology and clinical covariates, using a prespecified model and every attempted assay. Among the prespecified cases and the FDA authorised companion-diagnostic list (last updated 5 February 2026; accessed 26 July 2026), we identified no United States oncology companion diagnostic that directly measures substrate-specific degradation rate, half-life or flux (U.S. Food and Drug Administration, 2025c). That observation is scoped: it does not speak to laboratory-developed tests, exploratory trial assays, non-United States products or later authorisations.

Table 2 shows where ten representative cases stop, without collapsing mechanism, assay, use and product status into a single maturity score. Figure 1 defines the biological and measurement chain needed to interpret a protein-fate claim; Figure 3 addresses the subsequent, use-specific evidence routes once an assay is specified, separating drug-development use, clinical-test validation and testing-strategy utility. Status is tied to the individual case, the jurisdiction and the 26 July 2026 cut-off.

**TABLE 2:** Where representative cases stop between mechanism, measurement and use.

Case and context	Direct causal fate evidence	Patient-facing readout and fate calibration	Intended-use evidence and current status	Boundary and decisive next test
Endogenous degradation dysregulation				
SPOP–BRD4 in prostate versus endometrial models	Prostate-cancer *SPOP* variants impaired BRD4 degradation; endometrial variants increased BET-protein loss in the models tested	*SPOP* genotype plus BRD4 immunoreactivity is a patient-tissue abundance readout that has not been calibrated to BRD4 kinetics	Exploratory patient readout; candidate intended use not evaluated	Direction cannot transfer across allele, substrate or lineage. Calibrate matched tumour-tissue BRD4 state to endogenous kinetics and test a treatment interaction externally
FBXW7–MYC or NOTCH, trans reader failure	Independent studies support phosphodegron-dependent MYC loss; engineered NOTCH1 and tumour-derived *FBXW7* variants separate cis from trans failure in stated systems	Genotype, MYC or NOTCH abundance and pathway output remain substrate-specific contextual measures	Candidate intended use not evaluated	An *FBXW7* alteration does not name the operative substrate. Measure endogenous substrate kinetics in patient-derived, alteration-matched models and bridge the selected readout
*CTNNB1*–β-catenin, cis escape	β-TrCP/FWD1 recognition, ubiquitylation and radiolabelled decay support phosphodegron-dependent β-catenin loss; exon-3 reporter effects vary by allele	*CTNNB1* exon-3 genotype plus phospho-, total or nuclear β-catenin in tumour tissue	Candidate intended use not evaluated	Reporter signalling and static β-catenin do not measure turnover. Establish allele-resolved endogenous kinetics and specimen calibration before testing a defined decision
KEAP1–*NFE2L2*–CUL3, motif escape versus pooled states	ETGE/DLG recognition and selected *NFE2L2* motif variants support cis escape and longer apparent NRF2 half-life in defined models	KEAP1, *NFE2L2* and *CUL3* genotype with NRF2 abundance, localisation or target transcription is a composite non-kinetic state	Mechanism supported for stated motif variants; candidate intended use not evaluated for pooled pathway states	Evidence for selected *NFE2L2* variants cannot assign one fate to KEAP1, CUL3 and non-genetic states. Test allele- and context-specific kinetics with controlled redox and specimen handling
VHL–HIF, engineered HIF-1α evidence versus patient state	Engineered HIF-1α Pro564 and VHL perturbations support hydroxylation-dependent recognition, ubiquitylation and loss in defined systems	*VHL* genotype with hydroxy-HIF, HIF-1α or HIF-2α abundance is a cross-sectional package	Candidate intended use not evaluated	HIF-1α evidence does not transfer to HIF-2α or patient tumours. Test isoform-specific kinetics in patient-derived material with oxygen and timing controlled
URI1–MDM2–p53 in *APC*-deficient, *TP53*-wild-type colorectal models	URI1 increased MDM2-dependent p53 ubiquitylation and shortened apparent p53 half-life in the stated models	URI1 and p53 staining in human tissue records abundance, not patient p53 kinetics	Exploratory patient readout; candidate intended use not evaluated	No p53 degron was mapped and URI1 was not established as an E3. Reproduce causal kinetics in patient-derived *TP53*-wild-type material and calibrate a proximal tissue readout
USP17L2–BRD4 in prostate-cancer systems	USP17L2 bound and deubiquitinated BRD4; depletion shortened its apparent half-life and catalytic rescue restored the effect	USP17L2 and BRD4 abundance or IHC is a static patient-tissue package	Candidate intended use not evaluated	No analytically specified assay, treated-patient interaction or external validation. Test a calibrated BRD4 state in a prespecified BET-inhibitor cohort
Drug-induced degradation				
Lenalidomide–CRBN–IKZF in myeloma	Drug-bound CRBN recruitment and loss of IKZF1 and IKZF3 are supported independently in myeloma models	Exposure-timed peripheral-blood Aiolos loss is an exploratory pharmacodynamic precedent from mezigdomide, not a transferable lenalidomide assay	Lenalidomide-specific patient readout not evaluated here	Drug, CRBN state, neosubstrate, tissue and exposure must be matched. Build a lenalidomide-specific patient bridge and test whether the result changes a prespecified use
Vepdegestrant–CRBN–ERα, paired with plasma *ESR1* selection	CRBN-dependent ERα loss is supported in ER-positive breast-cancer models	A secondary report of exploratory exposure-timed ER abundance is distinct from the authorised Guardant360 companion diagnostic (CDx) plasma *ESR1* genotype assay	FDA-supported clinical validity for the exact labelled United States CDx use; device and therapeutic product authorised on 1 May 2026	*ESR1* genotype does not measure ER degradation. The retrospective final-device bridge is separate from a fate bridge and from incremental-net-benefit evidence
Adjacent pathway inhibition				
Belzutifan–HIF-2α	Not applicable. Belzutifan blocks HIF-2α dimerisation with HIF-1β (ARNT) and is not an induced degrader	The United States indications reviewed through 26 July 2026 did not use a VHL/HIF protein-fate companion diagnostic	United States therapeutic-product status only, current to 26 July 2026; fate-test intended use not evaluated	Do not infer assay status from product approval or transfer HIF-1α fate evidence. Any VHL/HIF assay requires a separate isoform-, specimen-, time- and use-specific bridge

Representative sources by row: SPOP–BRD4 (Dai et al., 2017; Zhang et al., 2017; Janouskova et al., 2017); FBXW7–MYC/NOTCH (Welcker et al., 2004; Yada et al., 2004; Welcker et al., 2022; O'Neil et al., 2007); CTNNB1–β-catenin (Amit et al., 2002; Kitagawa et al., 1999; Krishna et al., 2026); KEAP1–NFE2L2–CUL3 (Zhang et al., 2004; Tong et al., 2006; Tong et al., 2007; Fukutomi et al., 2014; Shibata et al., 2008; Shibata et al., 2009); VHL–HIF (Tanimoto et al., 2000; Ivan et al., 2001; Jaakkola et al., 2001; Chowdhury et al., 2016; van Leeuwaarde et al., 2025; Prowse et al., 1997; Cancer Genome Atlas Research Network, 2013); URI1–MDM2–p53 (Herranz-Montoya et al., 2025); USP17L2–BRD4 (Jin et al., 2018); lenalidomide–CRBN–IKZF (Fischer et al., 2014; Krönke et al., 2014; Lu et al., 2014; Richardson et al., 2023); vepdegestrant–CRBN–ERα/ESR1 (Campone et al., 2025; DailyMed, 2026; Gough et al., 2024; U.S., food and drug administration, 2026a; U.S., food and drug administration, 2026b; U.S. F ood and Drug Administration, 2026); belzutifan–HIF-2α (Dail yMed, 2026; U.S., food and drug administration, 2025a; U.S., food and drug administration, 2025b; U.S., food and drug administration, 2025c; U.S., food and drug administration, 2023b).

Cases are contrastive and not exhaustive. Mechanistic support is restricted to the stated experimental models; it does not establish clinical validation. Status is jurisdiction- and date-specific, with a cut-off of 26 July 2026. The ESR1 selector is genomic and does not measure ER, degradation.

**FIGURE 3:**
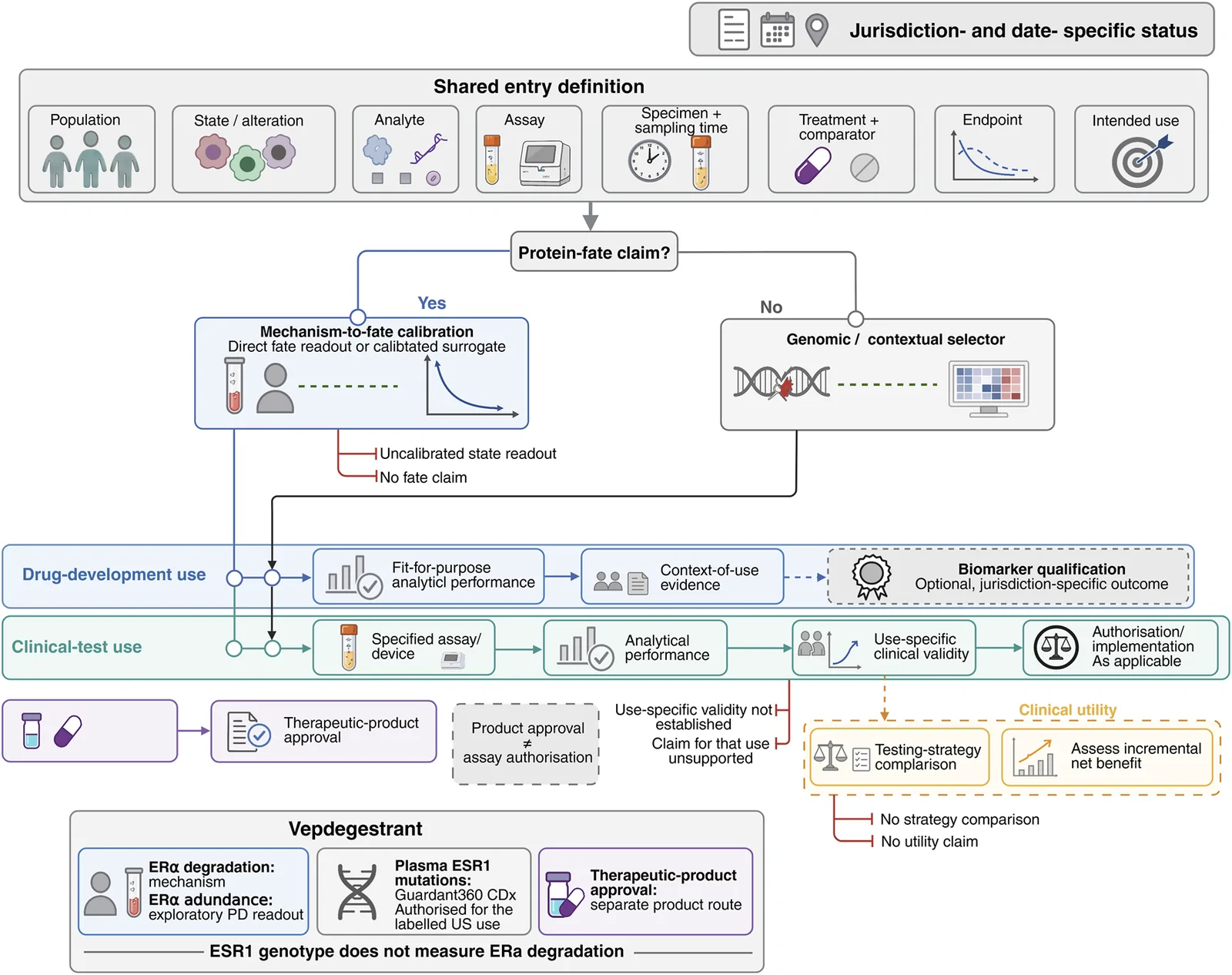
Non-interchangeable routes from an analytically specified measurement to clinical use. The shared entry defines population, biological state or alteration, analyte, assay, specimen, sampling time, treatment and comparator, endpoint and intended use. The drug-development lane requires fit-for-purpose analytical performance and evidence for its stated context of use. A mechanism-to-fate bridge is added when the interpretation claims degradation, half-life or flux; a genomic selector that makes no fate claim does not require this calibration. The clinical-test lane specifies the device or assay, establishes analytical performance and adds use-specific clinical evidence before any jurisdiction-specific authorisation or implementation status is assigned. Biomarker qualification and device authorisation remain distinct outcomes. Clinical utility or incremental net benefit forms a separate evidence branch because it depends on a comparison of testing strategies and is not automatically supplied by clinical validity. Therapeutic-product approval is also separate and confers neither assay authorisation nor biomarker qualification. Arrows represent evidentiary routes, not a universal sequence that every measurement must traverse in that jurisdiction.

Small changes can move the ground. A change in specimen, fixation, antibody, peptide, variant scope, laboratory, scoring or timing can alter analytical performance and the population the result applies to (Carr et al., 2014; Goldsmith et al., 2024). The earlier Guardant plasma application is useful platform precedent because its evidence can be inspected, not because it grants the later vepdegestrant use by inheritance (U.S. Food and Drug Administration, 2023a; U.S. Food and Drug Administration, 2026a; U.S. Food and Drug Administration, 2026b). Evidence has to be renewed at the level a change actually affects. Platform validation cannot be assumed for a new analyte; a trial assay cannot be equated with the final device; and therapeutic approval does not authorise a test. A measurement may still serve drug development even when no clinical-test use is proposed for it.

Authorisation and implementation should likewise hold on to the tested device version, laboratory setting and decision rule. A later technical change can be minor analytically yet material to clinical evidence, if it reclassifies patients. The resulting bridge should report both the concordance and the fate of the discordant cases, including whether those cases were even represented in the relevant efficacy population. Each change reopens the evidence at the level it touches.

## 7 Discussion: decisive experiments and falsifiers

Across the cases reviewed, mechanistic feasibility is more developed than clinical validation; improved patient stratification or treatment decisions from a protein-fate assay have not been demonstrated by the evidence presented here. Three questions therefore determine the appropriate claim. Does the tumour event alter the endogenous fate of the named substrate? Can that change be measured reproducibly in the intended specimen and sampling window? Does the result improve a clinical decision beyond genotype, pathology and routine clinical variables? Failing at a later stage narrows the claim without erasing valid mechanistic evidence. A strong mechanism cannot rescue an unmeasurable specimen state, and an analytically robust state does not, by itself, supply decision value without an appropriate comparison.

Patient-derived causal calibration should start in matched viable material that keeps lineage, allele and substrate context intact. Direct kinetics should be paired with cis repair or reconstruction, with perturbation of the proposed reader, E3 or deubiquitinase, and with rescue or epistasis under appropriate expression controls. Orthogonal methods should agree on the direction of change. The proposed route is rejected in three situations: if endogenous kinetics stay unchanged after repair; if the fate difference persists once the nominated component is removed; or if the effect vanishes in the native context. Findings should not be carried across *SPOP* lineages (Dai et al., 2017; Zhang et al., 2017; Janouskova et al., 2017), *FBXW7* substrates or *CTNNB1* alleles (Welcker et al., 2022; O'Neil et al., 2007; Amit et al., 2002; Kitagawa et al., 1999; Krishna et al., 2026), *NFE2L2* motif variants (Shibata et al., 2008; Shibata et al., 2009), or HIF isoforms (Ivan et al., 2001; Jaakkola et al., 2001; Chowdhury et al., 2016) without direct testing.

Treatment studies need exposure-aligned serial sampling, with the timing chosen for the drug, the tissue and the ethical setting. A baseline and an early on-treatment sample can establish an initial pharmacodynamic change. A later sample can tell maintained loss from recovery. Interruption, dose-change or progression samples are informative when they are obtained clinically, and pharmacokinetics has to accompany the protein result. Failed loss directs attention to engagement and E3 competence. Recovery directs attention to exposure and possible resynthesis. Downstream escape despite maintained loss directs attention to bypass signalling (Richardson et al., 2023; DailyMed, 2026; Gough et al., 2024; Robert et al., 2020; Zhu et al., 2019). Each of these interpretations has to be tested — a single fall in abundance establishes neither half-life, nor prediction, nor clinical utility.

Before any external outcome analysis, the candidate model should be prespecified and compared with genotype, routine pathology and the relevant clinical covariates. The denominator should include every specimen sought and every assay attempted. Missing specimen, insufficient tumour, analytical failure, indeterminate result and biological non-detection each need a separate code, because they imply different failures. Calibration, discrimination and decision consequences should be reported for the intended use, not inferred from a selected evaluable subset. A fate component is retained only when it gives reproducible, decision-relevant information that the simpler model lacks. Otherwise the protein measurement stays limited to its demonstrated mechanistic or pharmacodynamic use (National Cancer Institute, 2026; Goldsmith et al., 2024; U.S. Food and Drug Administration, 2019).

The evidentiary threshold rises with the intended use. Directional rescue may support a mechanistic claim. Specimen transfer asks for reproducible calibration and an interpretable failure rate. Prediction asks for prespecified treatment-effect modification. Utility asks for a comparison of testing strategies. Multiplicity and incomplete sampling should be handled before the decisive data are examined. Failure, too, is claim-specific: an unsuccessful rescue rejects the proposed route; failed calibration removes the kinetic interpretation; absent incremental performance excludes the protein measure from the decision model. A narrower, already demonstrated use can still stand (National Cancer Institute, 2026; Goldsmith et al., 2024; U.S. Food and Drug Administration, 2019).

Three limitations follow from the design itself. First, contrastive selection favours circuits with reasonably resolved mechanisms and visible patient interfaces, so it cannot estimate prevalence or the maturity of the wider field. Second, publication and availability are asymmetric — positive mechanistic findings and successful assay development are simply more visible than failed calibration or null validation. Third, recent clinical programmes may appear as protocols, registries or official records before full peer-reviewed reporting. We therefore avoid pooled estimates, keep source attribution, and restrict conclusions to the tested model, analyte, specimen, design and date. The framework should be re-examined in the circuits we left out, and against negative calibration studies as they appear.

## 8 Conclusion

Protein-fate measurement may add value when it resolves a decision-relevant state that simpler markers miss, but this advantage requires clinical validation. The appropriate biomarker depends on the biological question, not on proximity to a degradation mechanism. Mechanistic plausibility points to the substrate and the direction to test, but translation needs more: a calibrated, patient-compatible readout, a fully specified assay, and evidence for the stated use. A genomic selector can support a degrader without ever measuring degradation, as the plasma *ESR1* example shows. A protein measurement, equally, gains no special standing merely from sitting close to an E3–substrate mechanism. Its value has to be shown against what genotype, pathology and clinical context already provide.

Two testable hypotheses deserve priority. The first asks whether distinct upstream lesions converge on a substrate state that DNA categories miss. The second asks whether serial protein measurements can distinguish productive target loss, failed loss, recovery and downstream escape during therapy. These priorities are neither exhaustive nor presumptively superior to others. Both need calibration in patient-derived material and external comparison with simpler models. Failure can still be useful: it shows where translation breaks down, while leaving a narrower mechanistic or pharmacodynamic use intact, provided its claim, specimen and timing boundary stay explicit and supported.
